# Editorial: Public health in the context of life-limiting illnesses: patient-centered care in advanced and life-limiting illnesses

**DOI:** 10.3389/fpubh.2024.1454805

**Published:** 2024-07-22

**Authors:** Mevhibe B. Hocaoglu, Richard J. Siegert, Margaret Sandham, Rebecca J. Jarden, Rachel Chambers, Irene J. Higginson

**Affiliations:** ^1^Cicely Saunders Institute of Palliative Care, Rehabilitation and Policy, King's College London, London, United Kingdom; ^2^School of Clinical Sciences, Auckland University of Technology, Auckland, New Zealand; ^3^School of Psychology, Te Kura Hinengaro, Massey University, Auckland, New Zealand; ^4^Department of Nursing, Melbourne School of Health Sciences, The University of Melbourne, Carlton, VIC, Australia; ^5^Austin Health, Heidelberg, VIC, Australia; ^6^King's College Hospital, National Health Service (NHS) Trust, London, United Kingdom

**Keywords:** palliative care, public health, advanced illness, multimorbidity, life-limiting conditions, patient-centered outcomes

## Introduction

As global populations age and chronic diseases become more prevalent, public health systems face an increasing need for palliative and end-of-life care. Approximately 60% of individuals dying have prolonged advanced illnesses, necessitating comprehensive strategies to address their complex needs (1). Palliative care, characterized by its holistic and person-centered approach, is becoming an essential component of public health (2).

The COVID-19 pandemic has underscored the critical role of palliative care (3), revealing the intricate link between public health, health promotion, and palliative care services. This editorial explores how public health and palliative care intersect in the context of life-limiting illnesses, highlighting patient-centered care and complex symptom management as two fundamental aspects that palliative care offers.

## The growing demand for palliative care

The rising number of individuals living with advanced and life-limiting illnesses represents a significant public health challenge. This trend, driven by demographic shifts and the increasing prevalence of chronic conditions, demands a robust palliative care infrastructure (4). Palliative care aims to alleviate symptoms, manage pain, and improve quality of life for patients with serious illnesses (5). It should be integrated early in the illness trajectory to provide comprehensive support (6, 7).

## Palliative care's role in public health

Palliative care is an integral part of public health, essential for developing and implementing comprehensive healthcare services. Public health strategies aim at population-level interventions to promote health, prevent illness, and improve outcomes. Integrating palliative care into these initiatives ensures accessibility, equity, and responsiveness to the needs of diverse populations. The COVID-19 pandemic highlighted the necessity of such integration, revealing healthcare system gaps and underscoring the need for a robust palliative care framework.

In their study on a home health monitoring and education program for complex chronic patients, Soldado-Matoses et al. demonstrate how the primary care nurse led program effectively reduced hospital admissions and emergency department visits. This highlights the pivotal role of primary care nurses in chronic disease management through advanced competencies, showcasing how such initiatives can enhance public health outcomes.

Harrop et al. underscore the urgency of integrating palliative care into public health frameworks. Their longitudinal study on UK residents bereaved between March 2020 and January 2021, revealed high levels symptoms of Prolonged Grief Disorder (PGD). This necessitates strengthened social and specialist support, improved bereavement policies, and enhanced preparedness for future pandemics. Integrating palliative care ensures accessible, equitable, and responsive care, crucial for improving the quality of life for those with advanced and life-limiting illnesses.

## Enhancing palliative care access

Despite its benefits, palliative care is underutilized due to several barriers, including lack of awareness, cultural misconceptions, and systemic healthcare issues. Population-level patient-reported outcomes are essential for addressing public health objectives in life-limiting illnesses. Daveson et al., in their case study informed by the Organization for Economic Co-operation and Development's Best-Practice Public Health Framework, illustrated the importance of collecting and analyzing patient-reported data to improve pain management and address equity issues in healthcare delivery.

Shen et al., in their cross-sectional study identified barriers to inpatient palliative care referral among metastatic gynecologic cancer patients, influenced by hospital size, region, and specific cancer types, highlighting disparities in access based on institutional and geographical factors. Effective palliative care interventions require community engagement. Leonard et al. in their article revealed that community-engaged approaches significantly improve person-centered outcomes. The end-of-life needs of Aboriginal and immigrant communities present unique challenges to conventional medical models. Their analysis identified the need for trusted relationships, cultural practices around end-of-life care, and language barriers. The “Compassionate Communities” model emerged as a potential solution to support culturally sensitive care, indicating the necessity for healthcare systems to adapt to diverse cultural contexts.

## Reframing palliative care

The COVID-19 pandemic as a global public health emergency highlighted the critical need for policy reforms to support palliative and end-of-life care. Bradshaw et al. identified integration within health and social care systems, digital inclusivity, workforce development, support for care home managers, and addressing disparities of esteem as key policy priorities to equip care homes with the resources, capacity, and expertise needed for high-quality palliative care.

Rehabilitation is an integral part of palliative care (7). Lai et al., in their study on tracheal, bronchus, and lung cancer emphasized the role of rehabilitation in palliative care, and need for comprehensive rehabilitation services. According to projections, the burden of tracheal, bronchus, and lung cancer will continue to rise, particularly among females, necessitating targeted rehabilitation interventions to manage the disease effectively throughout the patient lifecycle.

## Conclusions

Integrating palliative care into public health strategies is imperative to address the complex needs of individuals with advanced and life-limiting illnesses. As the global population ages and chronic conditions become more prevalent, the demand for palliative care will continue to grow. Public health initiatives must prioritize the development and implementation of accessible, equitable, and culturally responsive palliative care services. By adopting a holistic, patient-centered approach, healthcare systems can improve the quality of life for patients and their families, ensuring comprehensive care and support throughout the illness trajectory.

Research highlighted in this editorial underscores the multifaceted nature of palliative care and its critical role in public health. From community-engaged interventions to policy reforms and targeted rehabilitation services, to capture of population-level patient-reported outcomes, a comprehensive approach is necessary to meet the diverse needs of patients with life-limiting illnesses. Collaboration across disciplines and community engagement at all stages of care design and implementation can build a more resilient and responsive healthcare system, prioritizing the wellbeing of individuals facing advanced illness.

## Author contributions

MH: Conceptualization, Writing – original draft, Writing – review & editing. RS: Writing – original draft, Writing – review & editing. MS: Writing – original draft, Writing – review & editing. RJ: Writing – original draft, Writing – review & editing. RC: Writing – original draft, Writing – review & editing. IH: Supervision, Writing – original draft, Writing – review & editing.
